# Developing a Riboswitch-Mediated Regulatory System for Metabolic Flux Control in Thermophilic *Bacillus methanolicus*

**DOI:** 10.3390/ijms22094686

**Published:** 2021-04-28

**Authors:** Marta Irla, Sigrid Hakvåg, Trygve Brautaset

**Affiliations:** Department of Biotechnology and Food Sciences, Norwegian University of Science and Technology, 7034 Trondheim, Norway; marta.k.irla@ntnu.no (M.I.); sigrid.hakvaag@sintef.no (S.H.)

**Keywords:** *Bacillus methanolicus*, thermophile, methanol, lysine riboswitch, *pbuE* riboswitch

## Abstract

Genome-wide transcriptomic data obtained in RNA-seq experiments can serve as a reliable source for identification of novel regulatory elements such as riboswitches and promoters. Riboswitches are parts of the 5′ untranslated region of mRNA molecules that can specifically bind various metabolites and control gene expression. For that reason, they have become an attractive tool for engineering biological systems, especially for the regulation of metabolic fluxes in industrial microorganisms. Promoters in the genomes of prokaryotes are located upstream of transcription start sites and their sequences are easily identifiable based on the primary transcriptome data. *Bacillus methanolicus* MGA3 is a candidate for use as an industrial workhorse in methanol-based bioprocesses and its metabolism has been studied in systems biology approaches in recent years, including transcriptome characterization through RNA-seq. Here, we identify a putative lysine riboswitch in *B. methanolicus,* and test and characterize it. We also select and experimentally verify 10 putative *B. methanolicus*-derived promoters differing in their predicted strength and present their functionality in combination with the lysine riboswitch. We further explore the potential of a *B. subtilis*-derived purine riboswitch for regulation of gene expression in the thermophilic *B. methanolicus*, establishing a novel tool for inducible gene expression in this bacterium.

## 1. Introduction

Transcriptomic data are a source of ample information on different regulatory elements including putative riboswitches and promoters. Riboswitches are RNA *cis*-regulatory elements present in genomes of organisms that belong to all domains of life: bacteria, archaea and eukaryotes, and mostly prevalent in bacteria and archaea [1]. They are located in the 5′ untranslated region of mRNA, upstream of the genes whose expression they regulate. Riboswitches usually consist of two domains: an aptamer, which is a sensing domain, and an expression platform—a regulating domain [2]. Riboswitches allosterically regulate gene expression through binding of a small-molecule ligand to the aptamer domain causing a conformational change, which then results in modification of specific gene expression [2]. This can for example contribute to regulation of activity of metabolic pathways such as the biosynthesis of vitamins (e.g., riboflavin, thiamine and cobalamin) and the metabolism of methionine, l-lysine and purines [3]. The mechanisms of action of riboswitches frequently found in bacterial species include transcription termination, translation initiation, mRNA degradation, splicing or RNA interference, with transcription termination being the most frequent [4,5]. Transcription termination is a mode of action of lysine riboswitch present in *Bacillus subtilis*, whereas the *Escherichia coli*-derived lysine riboswitch is a dual-acting riboswitch which, in addition to modulating translation initiation, is involved in the control of initial mRNA decay [6,7,8,9]. An example of a riboswitch with an atypical mode of action is a *B. subtilis*-derived riboswitch located upstream of the *pbuE* gene, encoding a purine efflux pump. In adenine-deficient conditions, the riboswitch is in an ‘off’-state and prevents gene expression by introduction of transcription termination through formation of a large hairpin [10]. This structure composed of 22 base pairs serves as an intrinsic terminator [10]. Thanks to this property, the *pbuE* riboswitch can be used as a part of an inducible gene expression system. It can be activated by purine analogues that are close chemical variants of adenine, 2,6-diaminopurine (2,6-DAP), 2-aminopurine (2-AP), purine (P) and N6-methyladenine (MA) [10].

Riboswitches have multiple potential uses in drug development as targets for antibacterial compounds, and in biotechnology as a tool for strain engineering. Popular engineering strategies involve the use of riboswitches to regulate the expression of the genes in the biosynthetic pathways competitive to the one of the chosen product [11,12], to deactivate riboswitch-controlled feedback inhibition in selected biosynthetic pathways [13] or to achieve dynamic control of the parts of biosynthetic pathways that can constitute metabolic burden for the cells, for example membrane proteins such as product transporters [14]. The desired riboswitches can either be detected through screening of sequences selected based on transcriptomic and functional studies [12,15], or designed using screening [2,11,16,17] or based on the pre-existing data [13].

Use of riboswitches for the fine-tuned regulation of gene expression is a promising prospect in strain engineering. In addition, access to repertoires of different promoters is fundamental for metabolic engineering of production strains. A common approach towards identification and characterization of novel promoters is based on screening of libraries of synthetic promoters, whose sequences are either completely randomized or partially based on consensus promoter sequences [18,19,20,21]. An exciting possibility for promoter engineering has emerged with development of systems biology technologies that include analysis of genomes, transcriptomes, proteomes and metabolomes. The primary transcriptome data provide information about transcription start sites and can be used for prediction of promoter sequences. This, in combination with information about transcripts abundancies, is a basis for determination of promoter strength and thus for a rational design of regulatory elements. In fact, this approach has been successfully used for identification and characterization of novel promoters in various bacterial species in recent years [18,22,23,24,25,26].

*Bacillus methanolicus* is a thermophilic bacterium with the potential of becoming an industrial workhorse for biotechnological production of value-added chemicals [27]. The wild-type MAG3 strain can naturally overproduce l-glutamate in methanol-controlled fed-batch fermentations, with final titers up to 60 g L^−1^ [28,29]. This feature is especially interesting as amino acids currently constitute most compounds produced in bioprocesses [30]. The possibility of establishing environmentally-friendly bioprocesses for production of bulk chemicals with the use of *B. methanolicus* as a host organism has spiked the interest of the scientific community in this bacterium and led to developments in understanding its metabolism, including characterization of its transcriptome [31,32,33,34,35]. Based on the genomic and transcriptomic data of the methylotrophic *B. methanolicus*, novel regulatory elements, such as a mannitol-inducible promoter, were previously predicted and characterized [36]. Here, we identify a putative lysine riboswitch based on transcriptomic data from *B. methanolicus*, characterize it and use it in a gene regulation system. From the same data, we further identify and characterize 10 promoters of diverse strength. Moreover, we use previously described *B. subtilis*-derived *pbuE* riboswitch to control gene expression in *B. methanolicus* [10].

## 2. Results

### 2.1. Transcriptome Analysis for Discovery of Novel Regulatory Elements

As previously described, the primary and whole transcriptomes of *B. methanolicus* were characterized through sequencing of mRNA isolated from *B. methanolicus* samples collected during cultivations in 16 different conditions [37]. This allowed for creation of a series of databases that cover a wide range of transcriptomic features such as transcriptional organization of operons, mRNA abundances, presence of *cis*-regulatory RNA elements and novel transcripts.

**Figure 1:**
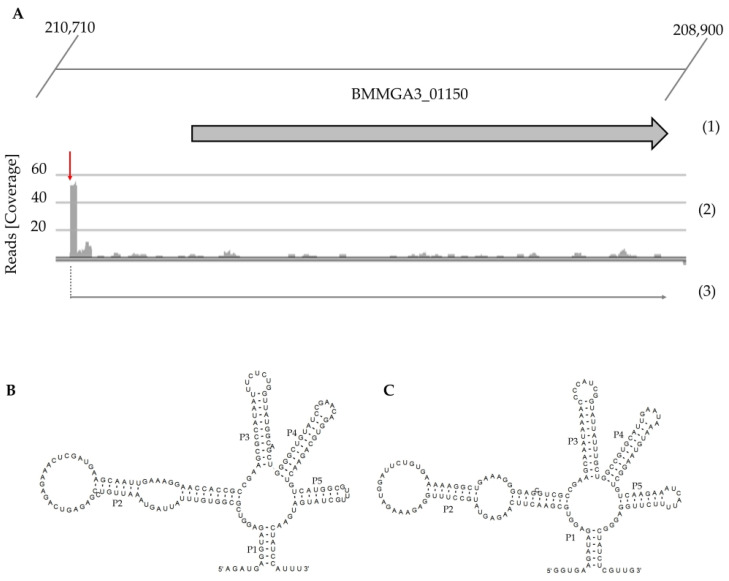
Transcriptional organization of putative lysine riboswitch of *B. methanolicus* MGA3 (**A**), its predicted structure (**B**) and predicted structure of *B. subtilis*-derived lysine riboswitch (**C**). (**A**) The first line (1) represents the genomic organization of the genes in the cluster, the next one (2) the mapped reads of 5′-end primary transcripts, and the last (3): the putative corresponding transcripts. The level of transcription is visualized with ReadXplorer [38] and given as absolute reads (coverage) at the corresponding genomic positions. Data are based on the previously published RNA-seq analysis [37]. (**B**) Predicted secondary structure of putative lysine riboswitch derived from *B. methanolicus*. (**C**) Predicted secondary structure of putative lysine riboswitch derived from *B. subtilis*. Both predicted structures were generated by R2DT using the lysine riboswitch template provided by Rfam [39,40].

It was predicted in that study that the 5′ untranslated region (5′ UTR) in mRNA belonging to the locus BMMGA3_01150 contains a putative lysine riboswitch. The gene located in locus BMMGA3_01150 in the genome of *B. methanolicus* encodes a putative amino acid permease similar to lysine permease YvsH from *B. subtilis* (51.64% identity), arginine:ornithine antiporter ArcD from *Pseudomonas aeruginosa* PAO1 (36.73% identity) and the lysine permease LysI from *Corynebacterium glutamicum* 13032 (27.77% identity). Rodionov et al. (2003) identified highly specific regulatory elements responsive to l-lysine upstream of the *yvsH* orthologs in different *Bacillus* species, e.g., *B. subtilis*, *B. cereus*, *B. halodurans*, *Geobacillus steorothemophilus*, which led them to suggest that YvsH is involved in the lysine transport in the above bacilli [37]. The sequence of 5′ UTR in the mRNA of locus BMMGA3_01150 was unraveled based on the primary transcriptome data which indicate the location of transcription start site (TSS) marked in Figure 1A with a red arrow.

Here, we study the transcript abundancies and the transcription start sites for respective transcripts to predict the sequence of the putative lysine riboswitch and to create a small set of promoters putatively differing in their strength, with a goal of detection and characterization of lysine riboswitches and novel promoters. Based on the available data for primary and whole transcriptome, 10 promoters differing in their strength were selected (Table 1). The promoter sequences were established using the location of the respective TSSs and the assumed strength using the information of transcripts abundancies expressed in LogRPKM unit [32].

To test the chosen promoters, the sequence upstream of the TSS in the methanol dehydrogenase (*mdh*) gene promoter (P*_mdh_*) in pTH1mp-*sfGFP* construct was replaced with the 200 bp sequences identified upstream of TSS of the genes with loci listed in Table 1. The resulting vectors were transformed to appropriate *B. methanolicus* hosts and the recombinant strains tested for fluorescence activity (Section 2.7).

### 2.2. Detection of Putative Lysine Riboswitch in the Genome of B. methanolicus

In the first stage of the lysine riboswitch analysis, the sequence of 5′ UTR of the gene in locus BMMGA3_01150 was compared to the Rfam database using nhammer tool that enables improved detection of remote DNA homologs. This comparison indicates that the sequence of the putative lysine riboswitch is: AGAUGAGGUAGAGGUCGCGGUGUUUAUUAGUAAAUUGUCCGAGAGUCAGAGAAACUCGAUGAAGCAAUUGAAAGGAACCACCGCCGAAGCGCCAUAAUUUCUCUGGUUAUGGCAGCUGGGGCUGUAUCCGAACAGGUGCAGAACUGUCAUGGCGUUUGCUAUGAUGAACUAUCCAUUU [39,42] and its secondary structure is similar to the lysine riboswitch structure derived from *B. subtilis* (Figure 1B,C) [39,40].

### 2.3. Construction of Model System to Characterize Riboswitches in B. methanolicus

**Figure 2:**
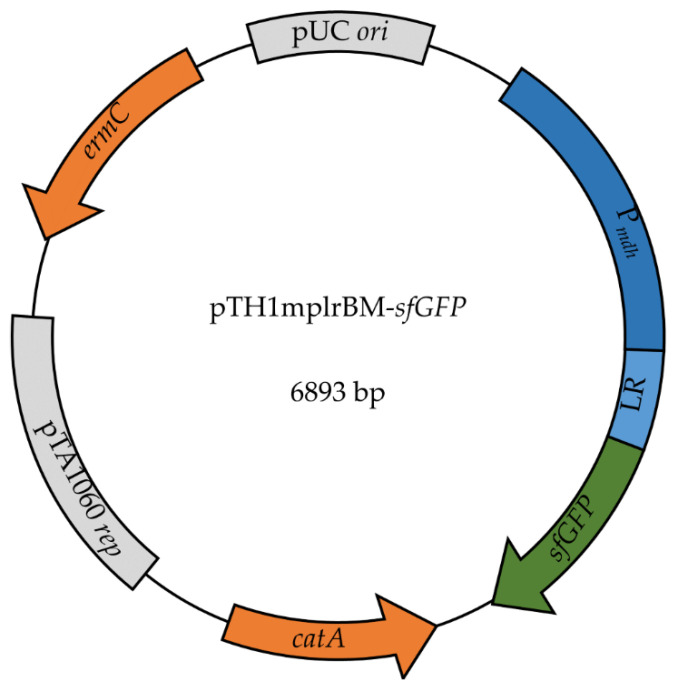
pTH1mp-*sfGFP*-based system for characterization of novel regulatory elements. Promoter P*_mdh_* controls *sfGFP* expression. For evaluation of lysine riboswitch, the 5′ UTR sequence of P*_mdh_* is replaced with 5′ UTR sequence that contains putative riboswitch.

To study the chosen riboswitches in *B. methanolicus*, an expression system, pTH1mp-*sfGFP*, composed of the reporter gene *sfGFP* under control of *mdh* promoter cloned intro a low-copy number rolling-circle vector derived from pHP13 was used. For testing of the riboswitches, the 5‘ UTR sequence of the *mdh* promoter was exchanged with the respective 5‘ UTR sequences that contained the analyzed riboswitches, as depicted in Figure 2.

In the case of the *B. methanolicus*-derived putative lysine riboswitch, the 5′ UTR of the gene in locus BMMGA3_01150 was used to replace the 5′ UTR in *mdh* promoter, and in this way plasmid pTH1mplrBM-*sfGFP* was created. The plasmid was then used to transform different hosts to tests lysine riboswitch functionality and characteristics in *B. methanolicus*, as well as mesophilic *B. subtilis* and *E. coli*.

### 2.4. Characterization of Functionality of Putative Lysine Riboswitch from B. methanolicus

**Figure 3:**
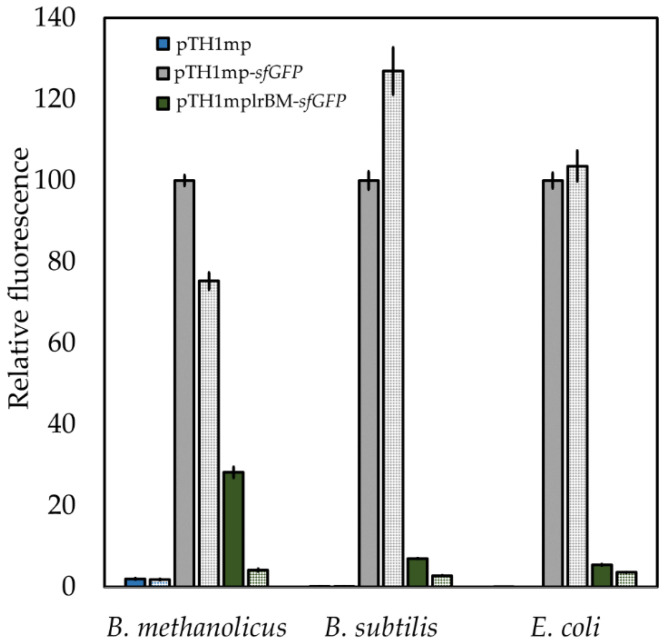
Relative mean sfGFP fluorescence in pellets of *B. methanolicus*, *B. subtilis*, or *E. coli* recombinant strains carrying empty vector (pTH1mp), a vector with *sfGFP* gene under the control of the P*_mdh_* (pTH1mp-*sfGFP*) or a vector with *sfGFP* gene under the control of the P*_mdh_* and lysine riboswitch (pTH1mplrBM-*sfGFP*). OD_600_ normalized fluorescence from strains carrying the empty vector and vector pTH1mp-*sfGFP* are used as controls. OD_600_ normalized sfGFP fluorescence from cultures without (solid bars) or with 20 mM lysine added (dotted bar) is shown. Relative fluorescence was calculated for each strain separately in relation to OD_600_ normalized fluorescence of strain carrying pTH1mp-*sfGFP* plasmid cultivated in medium supplemented with 20 mM l-lysine. The standard deviation of technical triplicates is shown. The raw data are available in supplementary information (Supplementary Table S2).

To study the effect of lysine on sfGFP fluorescence in bacterial strains with plasmids where *sfGFP* expression is regulated by *mdh* promoter (pTH1mp-*sfGFP*) or *mdh* promoter and *B. methanolicus*-derived lysine riboswitch (pTH1mplrBM-*sfGFP*), 20 mM l-lysine was added to the growth medium. A strain carrying the empty vector (pTH1mp) was used as control. The effect of l-lysine supplementations on the sfGFP fluorescence from different bacterial strains is shown in Figure 3. For all three bacterial strains (*B. methanolicus*, *B. subtilis* and *E. coli*), the sfGFP fluorescence decreased when l-lysine was added in the case where *sfGFP* expression is controlled by lysine riboswitch (pTH1mplrBM-*sfGFP* construct). For strains carrying the control vector (pTH1mp-*sfGFP*), the decrease in sfGFP fluorescence was either lower than for the strains carrying pTH1mplrBM-*sfGFP* or did not occur at all. Since the effect of l-lysine supplementation on sfGFP fluorescence in the strain carrying the control vector pTH1mp-*sfGFP* is visible only in one of the tested hosts, we attribute this effect to specific differences between the metabolism of the host organisms rather than to changes in the activity of *mdh* promoter. We additionally confirmed that the riboswitch responds exclusively to supplementation with l-lysine and no other amino acids tested (l-methionine, l-ornithine or l-proline). Some effect was shown due to supplementation of cadaverine; however, this was most probably due to the structural similarity between cadaverine and l-lysine (Supplementary Table S3). This means that the *B. methanolicus*-derived riboswitch is active in *B. methanolicus* in the presence of 20 mM l-lysine (Figure 3). We could also show that it is possible to functionally transfer a riboswitch derived from a thermophilic host to the mesophilic hosts *B. subtilis* and *E. coli.* To our knowledge, this is first report of transfer of a functional riboswitch derived from a thermophilic bacterium to mesophilic hosts. It should be noted that addition of l-lysine to the growth medium seems to affect growth rates and expression of *sfGFP* for all strains, including the control strain. However, the influence of the lysine riboswitch exceeds the physiological effect of the l-lysine supplementation.

### 2.5. Sensitivity of B. methanolicus-Derived Lysine Riboswitch in Its Native Host to Intracellular l-lysine Concentration

**Figure 4:**
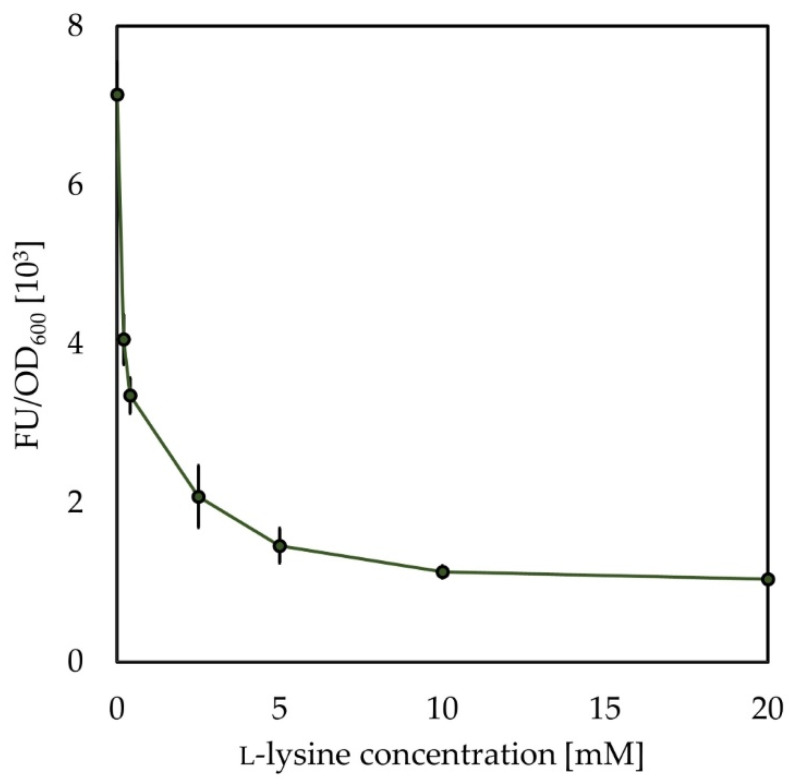
Effect of changes in concentrations of added l-lysine on OD_600_ normalized sfGFP fluorescence in pellets of recombinant strain *B. methanolicus* MGA3 (pTH1mplrBM-*sfGFP*). Mean sfGFP fluorescence in pellets of *B. methanolicus* recombinant strain carrying a vector with the *sfGFP* gene under the control of the P*_mdh_* and lysine riboswitch (pTH1mplrBM-*sfGFP*) is presented. The standard deviation of technical triplicates is shown.

To assess the sensitivity of the *B. methanolicus*-derived lysine riboswitch to changes in the l-lysine concentration, the titration of its activity was performed via the cultivation of the MGA3(pTH1mplrBM-*sfGFP*) strain, that carries the plasmid contacting the construct with its native lysine riboswitch, in a minimal medium supplemented with the following concentrations of l-lysine 0, 0.2, 0.4, 2.5, 5, 10, and 20 mM. As shown in Figure 4, the fluorescence intensity decreases with increasing concentrations of l-lysine. The 20 mM lysine is enough to fully inhibit expression of *sfGFP* in the tested strain.

### 2.6. Sensitivity of B. methanolicus-Derived Lysine Riboswitch in Its Native Host Extracellular l-lysine Concentration

**Figure 5:**
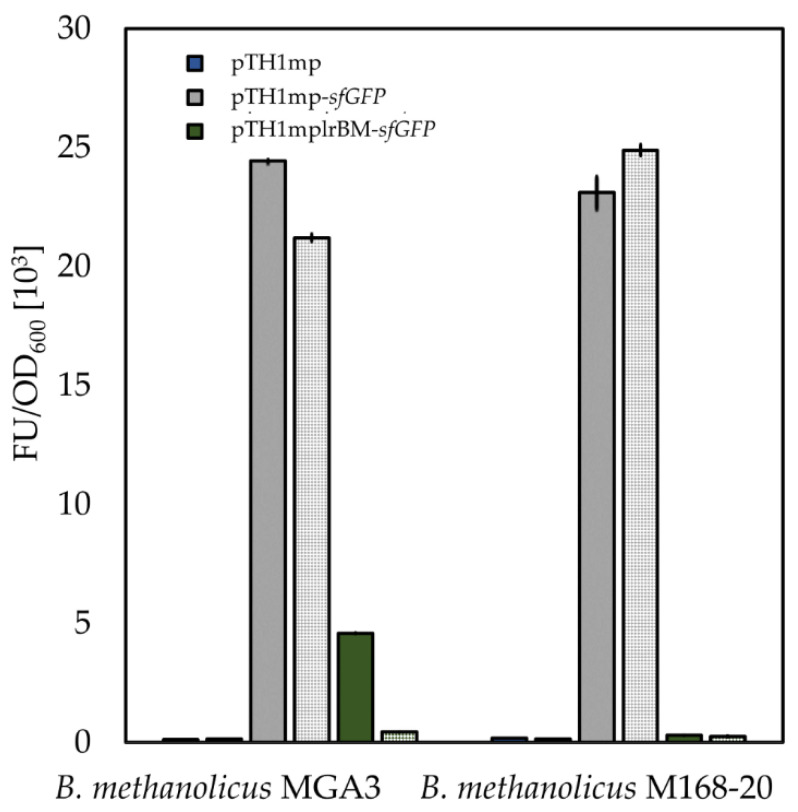
Effect of l-lysine production on activity of *B. methanolicus*-derived lysine riboswitch in wild-type *B. methanolicus* strain MGA3 and lysine overproducer strain M168-20. OD_600_ normalized mean sfGFP fluorescence in pellets of *B. methanolicus* recombinant strains is presented. Strains carrying the empty vector (pTH1mp) or a plasmid without the lysine riboswitch (pTH1mp-*sfGFP*) are used as controls. The strains carrying a vector pTH1mplrBM-*sfGFP* with *sfGFP* gene under the control of the P*_mdh_* and lysine riboswitch are used as experimental strains. Fluorescence without (solid bars) and with (dotted bars) supplementation of l-lysine is depicted. The standard deviation of technical triplicates is shown.

An important consideration is that the response of the lysine riboswitch depends strongly on the import rate of l-lysine into the bacterial cells. To better understand the response of the riboswitch to different l-lysine intracellular concentrations, we analyzed the fluorescence of sfGFP reporter protein, encoded by a lysine riboswitch-controlled gene in the wild-type *B. methanolicus* MGA3 strain and a l-lysine-overproducing strain M168-20. Both strains were transformed with the pTH1mplrBM-*sfGFP* plasmid and the fluorescence levels in each strain were evaluated. *B. methanolicus* strain M168-20 is a l-lysine-secreting classical mutant able to produce 140 mg L^−1^ of lysine during shake flask cultivations, which is approximately 20-fold higher than the wild-type strain MGA3 with l-lysine titers of 7 mg L^−1^ during flask cultivations [43,44,45]. As shown in Figure 5, when the M168-20 strain carrying the pTH1mplrBM-*sfGFP* plasmid was cultivated with or without addition of l-lysine to the medium, no sfGFP fluorescence was observed, indicating that the intracellular l-lysine concentration was high enough to completely arrest the expression *sfGFP* under control of lysine riboswitch. The differences in l-lysine secretion between wild-type and lysine-producing strains were confirmed experimentally and with l-lysine titers for analyzed strains presented in Supplementary Table S4. This finding suggests that the lysine riboswitch-based system can potentially be used as an indicator for the intracellular accumulation of l-lysine, and for regulation of gene expression and subsequent metabolic flux control in l-lysine-overproducing strains.

### 2.7. Characterization of Novel Promoters in B. methanolicus

**Figure 6:**
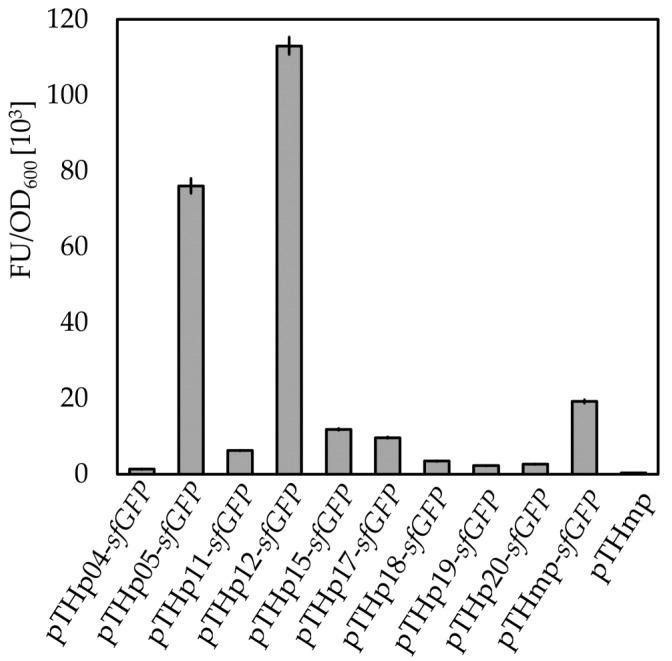
Experimental validation of strength of promoters predicted based on RNA-seq data. A set of putative promoters from *B. methanolicus* was cloned upstream of *sfGFP* gene. Mean OD_600_ normalized sfGFP fluorescence in pellets of recombinant strains of *B. methanolicus*, carrying *sfGFP* gene under control of different putative promoters is presented. Fluorescence from strains carrying either the empty vector (pTH1mp) or the vector pTH1mp-*sfGFP* with *sfGFP* under control of *P_mdh_* are used as control. The standard deviation of technical triplicates is shown.

By using the published transcriptomic data for *B. methanolicus*, we were able to identify a series of promoters differing in strength (Section 2.1; [32]). To experimentally validate the promoter strengths during methanol-based growth, we replaced the *mdh* promoter in pTH1mp-*sfGFP* plasmid with the selected promoters. The sequence of the ribosome binding site (RBS) was left unaltered to exclude any effect of the protein translation on the final sfGFP fluorescence. A total of 10 promoters with predicted different strengths were selected for further testing. However, the sequence predicted to be the strongest chromosome-based promoter (p01; P*_hps_*_-*phi*_) was not included due to technical issues with strain construction. As expected, the use of the selected promoters for control of *sfGFP* expression led to different levels of sfGFP fluorescence (Figure 6), either higher or lower than in the control strain MGA3 (pTH1mp-*sfGFP*), carrying the *mdh* promoter. However, since *mdh* is a plasmid-born gene in *B. methanolicus*, it is not possible to directly compare the transcriptomic data for that gene with the data on sfGFP fluorescence [46].

### 2.8. Consolidation of Identified Promoters and Lysine Riboswitch to Create Novel Tools for Gene Expression Regulation

**Figure 7:**
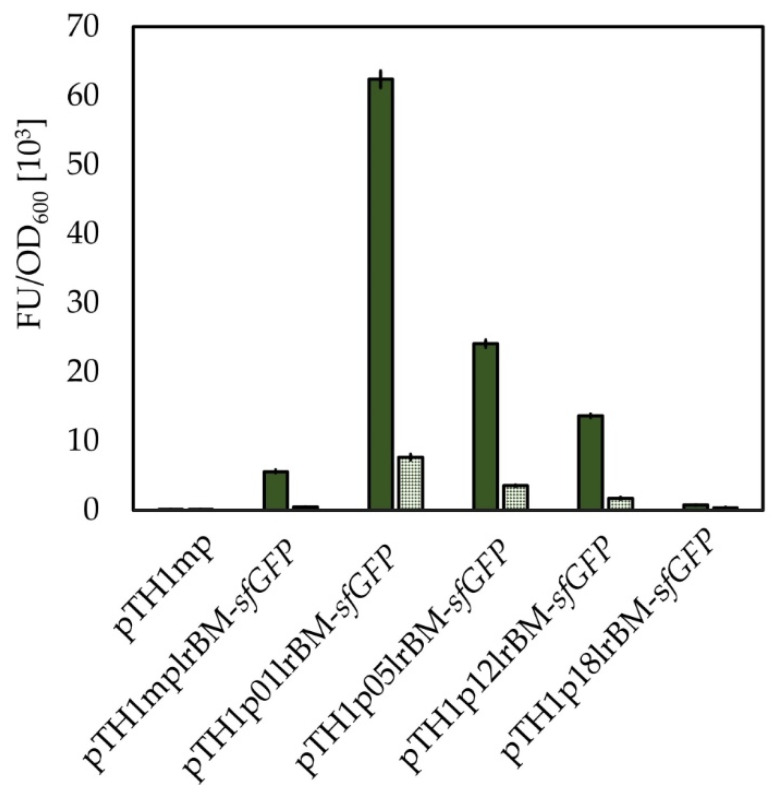
Influence of promoter strength on activity of lysine riboswitch. Figure presents OD_600_ normalized mean sfGFP fluorescence in pellets of *B. methanolicus* recombinant strains. *B. methanolicus* recombinant strains carry vector with *sfGFP* gene under control of different promoters. Fluorescence from strains carrying the empty vector (pTH1mp), or the vector with *sfGFP* gene under control of P*_mdh_* and lysine riboswitch (pTH1mplrBM-*sfGFP*) are used as control. Fluorescence from cultures with (dotted bars) or without (solid bars) 20 mM l-lysine added is shown. The standard deviation of technical triplicates is shown.

To expand the potential applicability of newly characterized lysine riboswitch and some of the tested promoters, we substituted the *mdh* promoter upstream of the lysine riboswitch. Based on the initial screening, four promoters were chosen to be used together with the lysine riboswitch for the control of *sfGFP* expression. Promoters p05 and p12 were included as promoters potentially stronger than *mdh* promoter, and promoter p18 as potentially weaker. We also included p01 (P*_hps-phi_*) which is predicted to be the strongest chromosomal promoter of *B. methanolicus* but was not able to experimentally validate this in this study. As shown in Figure 7, the background expression from the P*_hps-phi_* promoter is 7.3-fold stronger than from *mdh* promoter however, it also leads to higher translation readthrough when the riboswitch is activated by addition of l-lysine. Some discrepancies are observed between the strength of tested promoters when the riboswitch is present in the construct in comparison to constructs without the riboswitch. However, the general trend for the promoter strength showed that p05 and p12 are stronger than the *mdh* promoter and p18 is weaker. Here, we could show that the riboswitch-controlled gene expression can be additionally affected by the strength of the promoter located upstream of this regulatory element.

### 2.9. Transfer of Mesophilic Riboswitches to Thermophilic B. methanolicus

To further expand the genetic toolbox for *B. methanolicus* the new genetic system for riboswitch characterization (Section 2.3) was used to test the activity of *B. subtilis*-derived *pbuE* riboswitch in *B. methanolicus*. We also tested a variation of the *pbuE*-riboswitch with a modified P1 region, which can potentially lead to an increase in the sensitivity and dynamic range of the inducible system, as described in Marcano-Velazquez et al. (2019). The P1 region is a part of a purine riboswitch aptamer which forms a three-way RNA junction from helix P1 and hairpins P2 and P3, and changes in length of P1 helix affect the activity of *pbuE* riboswitch in the presence of 2-aminopurine [47]. Additionally, we have tested the *pbuE* riboswitch under the control of the P*_hps-phi_* promoter.

**Figure 8:**
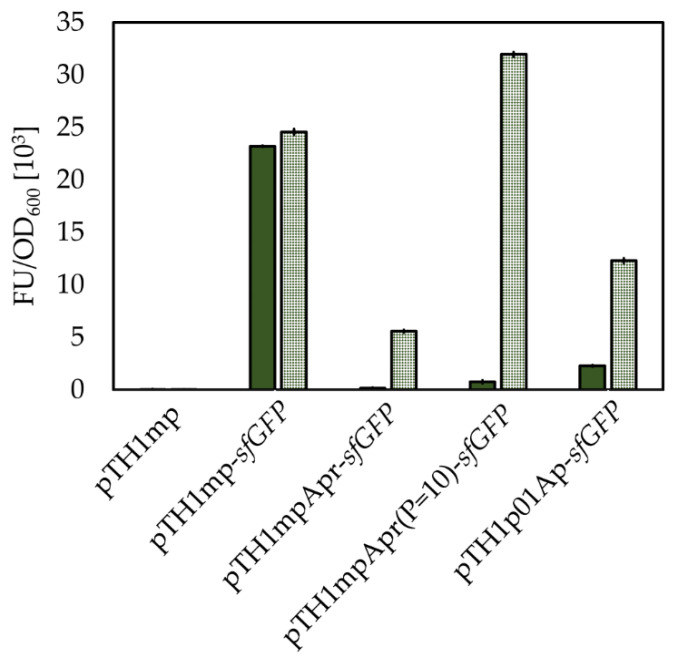
Activity of *B. subtlis*-derived *pbuE* riboswitch *in B. methanolicus*. Figure presents OD_600_ normalized mean sfGFP fluorescence in pellets of *B. methanolicus* recombinant strains: *B. methanolicus* recombinant strains carry vector with *sfGFP* gene under control of P*_mdh_* and *pbuE* riboswitch (pTH1mpApr-*sfGFP*), vector with *sfGFP* gene under control of P*_mdh_* and modified *pbuE* riboswitch (pTH1mpApr(P1 = 10)-*sfGFP*), vector with *sfGFP* gene under control of P*_hps-phi_* and *pbuE* riboswitch (pTH1p01Apr-*sfGFP*). Fluorescence from strains carrying the empty vector (pTH1mp) or a vector with the *sfGFP* gene under control of P*_mdh_* (pTH1mp-*sfGFP*) are used as control. Fluorescence from cultures with (dotted bars) or without (solid bars) 1 mM 2-aminopurine added is shown. The standard deviation of technical triplicates is shown.

As shown in Figure 8, the *pbuE* riboswitch is functional in *B. methanolicus*, and it is activated by supplementation of 1 mM 2-aminopurine into the growth media. Furthermore, both exchange of the promoter as well as the introduction of a mutation in the P1 helix have effect on the activity of the *pbuE* riboswitch. Exchange of the *mdh* promoter to the P*_hps-phi_* led to two changes in the activity of the riboswitch. Firstly, the background fluorescence of the reporter protein, sfGFP, increased which can be caused the fact that the P*_hps-phi_* promoter is putatively stronger than the *mdh* promoter. Secondly, in an activated state of the riboswitch—the sfGFP fluorescence was higher in the construct with a P*_hps-phi_* promoter in comparison to *mdh* promoter, this observation is could also be caused by the differences in promoter strength. The modification in the P1 region led to increased responsiveness of the riboswitch to supplementation with 2-aminopurine and slightly increased background in comparison to the wildtype version of the riboswitch. The use of *pbuE* riboswitch with P1 modification leads to relatively low background expressions together with adequately high expression in its fully induced state. In fact, when the modified variant of the *pbuE* riboswitch was used, the sfGFP fluorescence in its fully induced state was as high as the sfGFP fluorescence in strain containing control construct pTH1mp-*sfGFP*. Altogether, we were able to create a novel system for control of gene expression in *B. methanolicus*.

## 3. Discussion

In this study, a genetic system for investigation of properties of riboswitches in the thermophilic and methylotrophic *B. methanolicus* was created and used for characterization of a putative native lysine riboswitch and a *B. subtilis*-derived *pbuE* riboswitch. Based on the available transcriptomic data for *B. methanolicus* MGA3 the sequence of the putative lysine riboswitch was annotated and experimentally validated. It was shown to be functional in its native host and in two heterologous, mesophilic hosts. To our knowledge, this is a first example of functional transfer of the riboswitch from a thermophilic host to its mesophilic counterpart.

In order to fully activate the *B. methanolicus*-derived lysine riboswitch in its native host, l-lysine was supplemented to a final concentration of 5 mM (750 mg L^−1^) whereas *B. subtilis* and *E. coli*-derived riboswitches tested either in their native hosts or in *C. glutamicum* are activated by addition of 0.1 mM l-lysine or less [6,9,12]. This discrepancy in concentration of supplemented l-lysine can be caused either by differences in sensitivity of different riboswitches or by the activity of the l-lysine import system in different bacteria. The import and export system for l-lysine is not fully elucidated in *B. methanolicus* and it is, therefore, not possible to predict how the supplementation of growth medium affects the intracellular l-lysine concentrations in *B. methanolicus* [48]. For that reason, we tested the behavior of the lysine riboswitch in l-lysine-overproducing strain M168-20. Based on the available data for wild-type MGA3 and l-lysine-overproducing NOA2#13A52-8A66 strains, the intracellular l-lysine concentration in M168-20 can be assumed to lie within the range of 2.3–66.8 mM [29]. Our data show that this intracellular l-lysine concentration is enough to fully suppress expression of *sfGFP* under control of lysine riboswitch in M168-20 strain without additional supplementation of growth media with l-lysine. This result suggest that the *B. methanolicus*-derived riboswitch is less sensitive to changes in l-lysine concentrations than *E. coli* and *B. subtilis*-derived riboswitches which may be caused by the fact that the intracellular concentration of l-lysine in *B. methanolicus* is higher than in *B. subtilis* or *E. coli* [6,12,29,49].

Furthermore, a set of 10 different promoter sequences were identified based on transcriptomic data and experimentally verified for *B. methanolicus* in this study. Hitherto, only three promoters have been shown to be functional in *B. methanolicus*, one of them being *mdh* promoter. Methanol dehydrogenase constitutes up 22% of total soluble protein in cells of *B. methanolicus* grown under methanol-limiting conditions which makes it one of the most abundant proteins in the proteome of *B. methanolicus* [33,50]. The screening results of selected promoter sequences do not exactly reflect predictions made based on the transcriptomic data. This finding is not surprising, as the transcriptomic data were created based on the mRNA isolated from cells cultivated under different conditions, and the promoter activity in the current study was measured only in one of the conditions used for transcriptome analysis [37]. Altogether, the transcriptomic data are a useful guide for selection of active promoters based on the theoretical estimation of their strength, and it can be therefore used in future studies when a wide range of active promoters is needed. This approach for search of functional regulatory elements was shown to be successful before and seems to have some advantages over the use of libraries of randomized promoter sequences [18,22,23,24,25,26].

Promoters from the newly created collection were combined with the sequence of the characterized lysine riboswitch to establish a new tool for regulation of gene expression in *B. methanolicus*. Three promoters tested in this study were chosen, together with the untested promoter that regulates the expression of the operon encoding 3-hexulose-6-phosphate synthase (Hps) and phosphohexose isomerase (Phi) P*_hps-phi_* (p01). Based on the transcriptomic and enzymatic activity data, this promoter can be assumed to be strong in *B. methanolicus* [51,52]. The exchange of *mdh* promoter with other promoters had, in most of the cases, a predictable effect on the sfGFP fluorescence intensity; the use of promoters designated as p01 and p12 led to increased sfGFP fluorescence when the lysine riboswitch was not activated and use of p18 led to decreased fluorescence in comparison to *mdh* promoter. The residual (background) sfGFP fluorescence after l-lysine supplementation differed accordingly depending on the promoter strength, as it was higher for stronger promoters. This finding shows the flexibility and predictability of the lysine riboswitch-controlled system which will allow for its future use in more complex genetic systems.

Lastly, a system for regulation of gene expression based on the. *B. subtilis*-derived *pbuE* riboswitch was created and shown to be functional in *B. methanolicus*. This is in accordance with previous experiments, where it was active in different bacterial species, *E. coli*, *B. subtilis,* and thermophilic *Geobacillus thermoglucosidasius* and *Clostridium thermocellum* [53,54,55]. It was previously shown that genetic elements are transferable between mesophilic and thermophilic bacterial species, for example *B. megaterium*-derived xylose-inducible promoter drives gene expression in *B. methanolicus*. Furthermore, signal peptides derived from *G. stearothermophilus*, *B. subtilis* and *B. licheniformis* support protein secretion in *B. methanolicus* [41,56]. The *pbuE* riboswitch-based gene expression system has beneficial features for use in *B. methanolicus*. Firstly, 2-AP that is used for its activation is a gratuitous inducer, meaning that it does not serve as a carbon source for *B. methanolicus*. Furthermore, a *pbuE* riboswitch-based gene expression system is titratable which renders it useful for sensitive applications, such as expression of host toxic gene products. The properties of the system can be modified through exchange of the promoter upstream of *pbuE*-riboswitch. However, use of a strong promoter P*_hps_*_-*phi*_ was not beneficial in this case as it led to increased background. The change in sequence of P1 helix, which functions as the antitermination element of the *pbuE* riboswitch, led to improved properties for regulation of gene expression, as the sfGFP fluorescence upon full induction increased in comparison to wild-type lysine riboswitch, while remaining tunable, inducible and maintaining a low background expression.

The riboswitch-based systems developed here can potentially be used for the regulation of gene expression in *B. methanolicus*, with lysine riboswitches being especially useful for regulation of the genes that belong to the lysine biosynthesis pathway and 2-aminopurine riboswitch for inducible gene expression. Furthermore, use of regulated gene expression systems that are functional both in *E. coli* and *B. methanolicus* can become a solution to reoccurring cloning problems caused by utilization of a strong, constitutive promoter P*_mdh_*. High-level expression in *E. coli* is well studied and established, but expression of toxic genes especially requires special attention in order to avoid detrimental effects on the livability of *E. coli* cells [57]. Widely used approaches involve usage of pre-determined vector-host systems and control of plasmid copy number and tightly controlled promoters [58,59,60]. More conveniently, other approaches allow use of the vector of choice by utilizing different *E. coli* strains, reducing the copy number of ColE1-derived plasmids (CopyCutter EPI400 cells from Epicentre, Illumina, or ABLE C and K strains from Agilent), or by reducing incubation temperature. In the case of the *B. methanolicus*-derived riboswitch described in this study, expression of the gene of choice is strongly reduced in *E. coli* when placed under control of the riboswitch, allowing toxic genes/proteins to be cloned.

## 4. Materials and Methods

### 4.1. Strains, Plasmids, and Primers

Bacterial strains and plasmids used in this study are listed in Table 2, and primers (Sigma Aldrich) are listed in Supplementary Table S1. The *E. coli* strain DH5α was used as a general cloning host. The following strains were used as sources of gDNA for cloning of the lysine riboswitches: *B. subtilis* 168, *B. methanolicus* MGA3 and *E. coli* MG1655, and as hosts for screening of lysine riboswitches: *B. subtilis* 168, *B. methanolicus* MGA3. *B. methanolicus* M168-20 and *E. coli* DH5α.

### 4.2. Molecular Cloning

*E. coli* DH5α-competent cells were prepared according to the calcium chloride protocol, as described previously [62]. All standard molecular cloning procedures were carried out as described in Sambrook and Russell (2001) [63] or according to manuals provided by producers. Genomic DNA from *B. subtilis* 168, *B. methanolicus* MGA3 and *E. coli* MG1655 was isolated [64]. PCR products were amplified using Cloneamp HiFi PCR Premix (Takara) and purified using a QIAquick PCR Purification kit from Qiagen. DNA fragments were separated using 0.8% SeaKem LE agarose gels (Lonza) and isolated using a QIAquick Gel Extraction Kit (Qiagen). DNA fragments were joined by the means the isothermal DNA assembly [65]. Table 3 presents the list of DNA fragments that were used for plasmid construction this study. Colony PCR was performed using GoTaq DNA Polymerase (Promega). The sequences of cloned DNA fragments were confirmed by Sanger sequencing (Eurofins). *B. methanolicus* MGA3 and M168-20 were made electrocompetent and transformed by electroporation as described by Jakobsen et al. (2006) with modifications [56,66].

### 4.3. Media and Conditions for Shake Flask Cultivations

Recombinant *E. coli* and *B. subtilis* strains were cultivated at 37 °C in Lysogeny Broth (LB) or on LB agar plates [67] supplemented, when necessary, with 15 or 5 μg mL^−1^ chloramphenicol, respectively. For experiments involving lysine riboswitches, strains of *E. coli* and *B. subtilis* were cultivated at 37 °C in minimal medium MVcM supplemented with 10 g L^−1^ glucose, 20 mM l-lysine and 15 or 5 μg mL^−1^ chloramphenicol, respectively, unless stated otherwise. For standard cultivations, recombinant strains of *B. methanolicus* were cultivated in MVcM minimal medium with 200 mM methanol supplemented with 5 μg mL^−1^ chloramphenicol. MVcM medium contained the following, in 1 L of distilled water: K_2_HPO_4_, 4.09 g; NaH_2_PO_4_*H_2_O, 1.49 g; (NH_4_)_2_SO_4_, 2.11 g and was adjusted to pH 7.2 before autoclaving. MVcM medium was supplemented with 1 mL 1 M MgSO_4_*7H_2_O solution, 1 mL trace elements solution and 1 mL vitamin solution. The 1 M MgSO_4_*7H_2_O solution contained 246.47 g of MgSO_4_*7H_2_O in 1 L of distilled water, trace elements solution contained the following, in 1 L of distilled water: FeSO_4_*7H_2_0, 5.56 g; CuSO_4_*2H_2_O, 27.28 mg; CaCl_2_*2H_2_O, 7.35 g; CoCl_2_*6H_2_O, 40.50 mg; MnCl_2_*4H_2_O, 9.90 g; ZnSO_4_*7H_2_O, 287.54 mg; Na_2_MoO_4_*2H_2_O, 48.40 mg; H_3_BO_3_, 30.92 mg; HCl, 80 mL and vitamin solution contained the following, in 1 L of distilled water: biotin, thiamine hydrochloride, riboflavin, calcium d-pantothenate, pyridoxine hydrochloride, nicotinamide, 0.1 g each; 4-aminobenzoic acid, 0.02 g; folic acid, vitamin B_12_ and lipoic acid, 0.01 g each [52]. When needed, 10 g L^−1^ xylose (*v*/*v*) was added for induction. The 20 mM l-lysine or 1 mM 2-aminopurine were added when necessary. For precultures, minimal medium supplemented with 0.25 g L^−1^ yeast extract, designated MVcMY, was used. Cultivations were performed in triplicates in 250 mL baffled flasks (40 mL, 200 rpm, 50 °C), inoculated to a starting OD_600_ = 0.2. Growth was monitored by measuring OD_600_ with a cell density meter (WPA CO 8000 Biowave). Specific growth rates were calculated from the exponential phase, by calculating the slope of semi logarithmic plots of optical density versus time over a suitable period of time.

### 4.4. Fluorescence Microplate Assay

Bacterial strains were cultivated at either 37 or 50 °C in MVcM supplemented with 5 μg mL^−1^ chloramphenicol and 20 mM l-lysine for at least 6 h, reaching a minimum OD_600_ = 0.4 (one doubling) before harvesting. For fluorescence microplate assays, cells were harvested by centrifugation at 13,000 rpm for 10 min and washed twice with PBS, before resuspension in PBS. A volume of 200 μL of resuspended cells were used for measuring fluorescence in microtiter plates (Falcon™ 96-well, clear bottom black polystyrene Imaging Microplate). An *Infinite 200 PRO* plate reader (Tecan Group Ltd.) was used for fluorescence measurements, with settings: ex 485/9 nm, em 535/20 nm. sfGFP signals were collected with a gain setting of 90 and divided by OD_600_for normalization.

### 4.5. Determination of Amino Acid Concentration

For the analysis of amino acids concentrations, 1 mL of the bacterial cultures was centrifuged for 10 min at 13,000 g. Extracellular amino acids were quantified by means of high-pressure liquid chromatography (Waters Alliance e2695 Separations Module) after FMOC-Cl (fluorenylmethyloxycarbonyl chloride) derivatization of the supernatants, according to the protocol described before [68]. Samples were separated on a C18 column (Symmetry C18 Column, 100 Å, 3.5 µm, 4.6 mm × 75 mm, Waters) according to the gradient flow presented in Table 4 where A is the elution buffer (50 mM Na-acetate pH = 4.2) and B is an organic solvent (acetonitrile).

The detection was performed with a fluorescence detector (Waters 2475 HPLC Multi Fluorescence Detector), with excitation and emission at 265 and 315 nm, respectively.

## 5. Conclusions

In this report, we present the feasibility of riboswitch-based systems for control of gene expression in a thermophilic methylotroph, *B. methanolicus* MGA3. Based on the RNA-seq data, we have detected a putative lysine riboswitch in the genome of *B. methanolicus* and confirmed its functionality through analysis of changes in sfGFP fluorescence with and without the presence of l-lysine. The response of the lysine riboswitch to changes in intra- and extracellular concentrations of l-lysine *B. methanolicus* demonstrates its feasibility for potential use in metabolic engineering for metabolic flux control or as a lysine sensor. Furthermore, ten novel promoters were predicted based on RNA-seq data and here experimentally shown to be functional in *B. methanolicus*. The promoters differ in their strength, ranging from 0.1- to 5.9-fold of the activity of the control promoter, P*_mdh_*. A system for regulation of gene expression based on the *B. subtilis*-derived *pbuE* riboswitch was created and shown to be functional in *B. methanolicus*. Lastly, the genetic tools were used in combination, presenting the potential for creation of novel genetic systems for control of gene expression in *B. methanolicus.*

## Figures and Tables

**Table 1:** List of promoters chosen for characterization. The list contains the promoter designation used in this study, locus tag of the gene controlled by the promoter, the protein encoded by the gene and the transcript abundancies for the chosen promoters in the LogRPKM unit which normalizes the data per reads per kilobase of coding DNA sequence (CDS) per million mapped reads (RPKM) [41].

Promoter Designation	Locus Tag	Encoded Protein	LogRPKM
p01 (P*_hps-phi_*)	BMMGA3_06845- BMMGA3_06840	3-Hexulose-6-phosphate synthase and 3-hexulose-6-phosphate isomerase	12307.54
p04	BMMGA3_16050	Putative sugar phosphate isomerase, RpiB	3472.63
p05	BMMGA3_01940	Glutamate synthase, large subunit, GltA, small subunit, GltB	1779.97
p11	BMMGA3_14565	NH_3_-dependent NAD^+^ synthetase, NadE	1598.52
p12	BMMGA3_10275	Dihydroxy-acid dehydratase, IlvD	758.16
p15	BMMGA3_16310	Pyridoxine kinase, PdxK	231.45
p17	BMMGA3_11740	Hypothetical protein	160.00
p18	BMMGA3_02750	d-Isomer specific 2-hydroxyacid dehydrogenase NAD-binding protein	107.14
p19	BMMGA3_09580	Putative membrane protein	66.73
p20	BMMGA3_09845	Hypothetical protein	39.59

**Table 2:** Bacterial strains and plasmids used in this study.

**Strain Name**	**Relevant Characteristics**	**Reference**
*E. coli* DH5α	General cloning host, F-*thi*-1 *endA*1 *hsdR*17(r-,m-) *supE*44 _*lacU*169 (_80*lacZ*_M15) *recA*1 *gyrA*96 *relA*1	Stratagene
*E. coli* MG1655	Wild-type strain; F- λ- *ilvG*- rfb-50 *rph*-1	ATCC 47076
*B. methanolicus* MGA3	Wild-type strain	ATCC 53907
*B. methanolicus* M168-20	1st generation S-(2-aminoethyl) cysteine-resistant mutant of MGA3; l-lysine overproducer	[43]
*B. subtilis* 168	Wild-type strain	ATCC 23857
**Plasmid Name**	**Relevant Characteristics**	**Reference**
pTH1mp	Cm^R^; derivative of pTH1mp-*lysC* for gene expression under control of the *mdh* promoter. The *lysC* gene was replaced with multiple cloning site.	[36]
sfGFP-pBAD	Am^R^; pBAD/His derivative for expression of *sfGFP*	Addgene # 54519 [61]
pTH1mp-*sfGFP*	Cm^R^; pTH1mp derivative for expression of *sfGFP* from sfGFP-pBAD under control of the *mdh* promoter	This study
pTH1mplrBM-*sfGFP*	Cm^R^; pTH1mp derivative for expression of *sfGFP* from sfGFP-pBAD under control of the *mdh* promoter and *B. methanolicus*-derived lysine riboswitch	This study
pTH1p04-*sfGFP*	Cm^R^; pTH1mp derivative for expression of *sfGFP* from sfGFP-pBAD under control of the promoter p04 (Table 1)	This study
pTH1p05-*sfGFP*	Cm^R^; pTH1mp derivative for expression of *sfGFP* from sfGFP-pBAD under control of the promoter p05 (Table 1)	This study
pTH1p11-*sfGFP*	Cm^R^; pTH1mp derivative for expression of *sfGFP* from sfGFP-pBAD under control of the promoter p11 (Table 1)	This study
pTH1p12-*sfGFP*	Cm^R^; pTH1mp derivative for expression of *sfGFP* from sfGFP-pBAD under control of the promoter p12 (Table 1)	This study
pTH1p15-*sfGFP*	Cm^R^; pTH1mp derivative for expression of *sfGFP* from sfGFP-pBAD under control of the promoter p15 (Table 1)	This study
pTH1p17-*sfGFP*	Cm^R^; pTH1mp derivative for expression of *sfGFP* from sfGFP-pBAD under control of the promoter p17 (Table 1)	This study
pTH1p18-*sfGFP*	Cm^R^; pTH1mp derivative for expression of *sfGFP* from sfGFP-pBAD under control of the promoter p18 (Table 1)	This study
pTH1p19-*sfGFP*	Cm^R^; pTH1mp derivative for expression of *sfGFP* from sfGFP-pBAD under control of the promoter p19 (Table 1)	This study
pTH1p20-*sfGFP*	Cm^R^; pTH1mp derivative for expression of *sfGFP* from sfGFP-pBAD under control of the promoter p20 (Table 1)	This study
pTH1p01lrBM-*sfGFP*	Cm^R^; pTH1mp derivative for expression of *sfGFP* from sfGFP-pBAD under control of the promoter p01 (Table 1) and *B. methanolicus*-derived lysine riboswitch	This study
pTH1p05lrBM-*sfGFP*	Cm^R^; pTH1mp derivative for expression of *sfGFP* from sfGFP-pBAD under control of the promoter p05 (Table 1) and *B. methanolicus*-derived lysine riboswitch	This study
pTH1p12lrBM-*sfGFP*	Cm^R^; pTH1mp derivative for expression of *sfGFP* from sfGFP-pBAD under control of the promoter p12 (Table 1) and *B. methanolicus* derived-lysine riboswitch	This study
pTH1p18lrBM-*sfGFP*	Cm^R^; pTH1mp derivative for expression of *sfGFP* from sfGFP-pBAD under control of the promoter p18 (Table 1) and *B. methanolicus*-derived lysine riboswitch	This study
pTH1mpApr-*sfGFP*	Cm^R^; pTH1mp derivative for expression of *sfGFP* from sfGFP-pBAD under control of the *mdh* promoter and *B. subtilis*-derived *pbuE* riboswitch	This study
pTH1mpApr(P1 = 10)-*sfGFP*	Cm^R^; pTH1mp derivative for expression of *sfGFP* from sfGFP-pBAD under control of the *mdh* promoter and modified *B. subtilis*-derived *pbuE* riboswitch	This study
pTH1p01Apr-*sfGFP*	Cm^R^; pTH1mp derivative for expression of *sfGFP* from sfGFP-pBAD under control of the promoter p01 (Table 1) and *B. subtilis*-derived *pbuE* riboswitch	This study

**Table 3:** List of restriction enzymes used for linearization of vector backbones and primers for amplification of inserts used in construction of plasmids.

Plasmid Name	Vector Backbone and Method of Linearization	Insert and Primers Used for Amplification
pTH1mp-*sfGFP*	pTH1mp digested with *Xba*I and *Afl*III	*sfGFP* gene PCR-amplified with sfGFP-pTH1mp_FW and sfGFP-pTH1mp_RW
pTH1mplrBM-*sfGFP*	pTHmp-*sfGFP* PCR-amplified with PSGF and PSGR	Lysine riboswitch derived from *B. methanolicus* PCR-amplified with LRIF and LRIR
pTH1p04-*sfGFP*	pTHmp-*sfGFP* PCR-amplified with PROM01 and PROM02	Promoter p04 PCR-amplified with PROM09 and PROM10
pTH1p05-*sfGFP*	pTHmp-*sfGFP* PCR-amplified with PROM01 and PROM02	Promoter p05 PCR-amplified with PROM11 and PROM12
pTH1p11-*sfGFP*	pTHmp-*sfGFP* PCR-amplified with PROM01 and PROM02	Promoter p11 PCR-amplified with PROM23 and PROM24
pTH1p12-*sfGFP*	pTHmp-*sfGFP* PCR-amplified with PROM01 and PROM02	Promoter p12 PCR-amplified with PROM25 and PROM26
pTH1p15-*sfGFP*	pTHmp-*sfGFP* PCR-amplified with PROM01 and PROM02	Promoter p15 PCR-amplified with PROM31 and PROM32
pTH1p17-*sfGFP*	pTHmp-*sfGFP* PCR-amplified with PROM01 and PROM02	Promoter p17 PCR-amplified with PROM35 and PROM36
pTH1p18-*sfGFP*	pTHmp-*sfGFP* PCR-amplified with PROM01 and PROM02	Promoter p18 PCR-amplified with PROM37 and PROM38
pTH1p19-*sfGFP*	pTHmp-*sfGFP* PCR-amplified with PROM01 and PROM02	Promoter p19 PCR-amplified with PROM39 and PROM40
pTH1p20-*sfGFP*	pTHmp-*sfGFP* PCR-amplified with PROM01 and PROM02	Promoter p20 PCR-amplified with PROM41 and PROM42
pTH1p01lrBM-*sfGFP*	pTHmplrBM-*sfGFP* PCR-amplified with LR11 and PROM02	Promoter p01 PCR-amplified with PROM03 and LR12
pTH1p05lrBM-*sfGFP*	pTHmplrBM-*sfGFP* PCR-amplified with LR11 and PROM02	Promoter p05 PCR-amplified with PROM11 and LR20
pTH1p12lrBM-*sfGFP*	pTHmplrBM-*sfGFP* PCR-amplified with LR11 and PROM02	Promoter p12 PCR-amplified with PROM25 and LR14
pTH1p18lrBM-*sfGFP*	pTHmplrBM-*sfGFP* PCR-amplified with LR11 and PROM02	Promoter p18 PCR-amplified with PROM37 and LR15
pTH1mpApr-*sfGFP*	pTH1mp-*sfGFP* PCR-amplified with PGF2 and PSGR	*B. subtilis*-derived *pbuE* riboswitch amplified with APR01 and APR02
pTH1mpApr(P1 = 10)-*sfGFP*	pTH1mpApr-*sfGFP* PCR-amplified in site directed mutagenesis approach with APR05 and APR06	
pTH1p01Apr-*sfGFP*	pTH1mp-*sfGFP* PCR-amplified with PROM01 and PROM02	Promoter p01 PCR-amplified with PROM03 and APR03; *B. subtilis*-derived *pbuE* riboswitch amplified with APR04 and APR02

**Table 4:** Linear elution profile of HPLC method used for analysis of l-lysine concertation. A is the elution buffer (50 mM Na-acetate pH = 4.2) and B is an organic solvent (acetonitrile).

Time (min)	Total Flow	%A	%B
	1.3	62.0	38.0
5	1.3	62.0	38.0
12	1.3	43.0	57.0
14	1.3	24.0	76.0
15	1.3	43.0	57.0
18	1.3	620	38.0

## Supplementary Materials

The following are available online at https://www.mdpi.com/article/10.3390/ijms22094686/s1, Table S1: List of primers used in this study, Table S2: Relative mean sfGFP fluorescence in pellets of B. methanolicus, B. subtilis, or E. coli recombinant strains carrying empty vector (pTH1mp), vector with sfGFP gene under the control of the Pmdh (pTH1mp-sfGFP) and vector with sfGFP gene under the control of the Pmdh and lysine riboswitch (pTH1mplrBM-sfGFP). OD600 normalized fluorescence from strains carrying the empty vector and vector pTH1mp-sfGFP are used as controls. OD600 normalized sfGFP fluorescence from cultures without or with 20 mM lysine added is shown (raw data for Figure 3). Table S3. Effect of different amino acids on activity of lysine riboswitch. OD600 normalized sfGFP fluorescence from cultures without addition of amino acid or with 10 mM l-lysine, cadaverine, l-methionine, l-ornithine or l-proline is shown. The standard deviation of technical triplicates is shown. Table S4. L-Lysine final titers for MGA3 and M168-20 strains carrying different plasmids for controlled sfGFP production. The means of triplicates with standard deviations are shown.
