# Supplementary material for: Developing a Riboswitch-Mediated Regulatory System for Metabolic Flux Control in Thermophilic Bacillus methanolicus
Source: Int J Mol Sci. 2021 Apr 28;22(9):4686. doi: 10.3390/ijms22094686 (PMC8124236; doi:10.3390/ijms22094686)
Supplement: Supplementary file 1 [file ijms-22-04686-s001.zip › ijms-1161510-supplementary.pdf]

Supplementary information to

Developing a riboswitch-mediated regulatory system for metabolic flux control in thermophilic *Bacillus methanolicus*

Marta Irla[1], Sigrid Hakvåg[1], Trygve Brautaset[1]*

[1]Department of Biotechnology and Food Sciences, Norwegian University of Science and Technology, Trondheim, Norway

*Corresponding author: Trygve Brautaset
Phone: +47 73593315
Email: trygve.brautaset@ntnu.

Supplementary Table S1. List of primers used in this study.

Primer name	Sequence	Function
VJFW	TCTAATCCTTCTAAAAAATATAATTTAGAA AACTAAG	sequencing primer for pTH1mp: FW
VJRW	GGTGCGGGCCTCTTCGCTATTACG	sequencing primer for pTH1mp; RV
sfGFP-pTH1mp_FW	TAAATAGGAGGTAGTACATATGCGTAAAG GCGAAGAGC	pTH1mp-*sfGFP* was constructed by amplifying *sfGFP* from sfGFP-pBAD with primers sfGFP-pTH1mp_FW and sfGFP-pTH1mp_RW, and joining the resulting PCR product with XbaI and AflIII digested pTHmp by means of the isothermal DNA assembly method
sfGFP-pTH1mp_RW	GACCTATGGCGGGTACCATATCATTTGTAC AGTTCATCCATACC	pTH1mp-*sfGFP* was constructed by amplifying *sfGFP* from sfGFP-pBAD with primers sfGFP-pTH1mp_FW and sfGFP-pTH1mp_RW, and joining the resulting PCR product with XbaI and AflIII digested pTHmp by means of the isothermal DNA assembly method
PSGF	ATGCGTAAAGGCGAAGAGCTGTT	Forward primer for amplification of vector backbone pTHmp-*sfGFP*
PSGR	AAAACTAGTTTAAATGCTAATCTGGATGTT TGTC	Reverse primer for amplification of vector backbone pTHmp-*sfGFP*
LRIF	GATTAGCATTTAAACTAGTTTGTAGTTGA ATAATGATTCCTCATCAG	Forward primer for amplification of lysine riboswitches derived from *B. methanolicus* to create plasmid pTH1mplrBM-*sfGFP* through assembly with PCR-amplified vector backbone
LRIR	AGCTCTTCGCCTTTACGCATATTTCTTCTCC TCTCATGTT	Reverse primer for amplification of lysine riboswitches derived from *B. methanolicus* to create plasmid pTH1mplrBM-*sfGFP* through assembly with PCR-amplified vector backbone
PROM01	GTAAACAATTACATAAATAGGAGGTAGT	Forward primer for amplification of vector backbone pTHmp-*sfGFP* for constructs pTH1p05-*sfGFP*, pTH1p12-*sfGFP* and pTH1p18-*sfGFP*
PROM02	AATCTTTTGAAAATAAGCGAGAGAA	Reverse primer for amplification of vector backbone pTHmp-*sfGFP* for constructs pTH1p05-*sfGFP*, pTH1p12-*sfGFP* and pTH1p18-*sfGFP*
PROM09	TCGCTTATTTTCAAAAGATTTTTAAGCTATA AGCTCTTT	Forward primer for amplification of promoter (p04) derived from *B. methanolicus* to create plasmid pTH1p04-*sfGFP* through assembly with PCR-amplified vector backbone
PROM10	CTATTTATGTAATTGTTTACATATTATTGCA TAAAATG	Reverse primer for amplification of promoter (p04) derived from *B. methanolicus* to create plasmid pTH1p04-*sfGFP* through assembly with PCR-amplified vector backbone
PROM11	TCGCTTATTTTCAAAAGATTTAAAAAATAT TGAAGTTGTCTTA	Forward primer for amplification of promoter (p05) derived from *B. methanolicus* to create plasmid pTH1p05-*sfGFP* through assembly with PCR-amplified vector backbone
PROM12	CTATTTATGTAATTGTTTACAACTATTATTT TAGTTTTCAAAAAT	Reverse primer for amplification of promoter (p05) derived from *B. methanolicus* to create plasmid pTH1p05-*sfGFP* through assembly with PCR-amplified vector backbone

PROM23	TCGCTTATTTTCAAAAGATTAAAATTTGAA ATGGCAGCCACATTTT	Forward primer for amplification of promoter (p11) derived from *B. methanolicus* to create plasmid pTH1p11-*sfGFP* through assembly with PCR-amplified vector backbone
PROM24	CTATTTATGTAATTGTTTACATTTATCATTA TATCCTTTCTTAACTTTTCT	Reverse primer for amplification of promoter (p11) derived from *B. methanolicus* to create plasmid pTH1p11-*sfGFP* through assembly with PCR-amplified vector backbone
PROM25	TCGCTTATTTTCAAAAGATTAATCGCATTTT CTTCCATTA	Forward primer for amplification of promoter (p12) derived from *B. methanolicus* to create plasmid pTH1p12-*sfGFP* through assembly with PCR-amplified vector backbone
PROM26	CTATTTATGTAATTGTTTACCTTTCAAATAT AGACCTTTTCAC	Reverse primer for amplification of promoter (p12) derived from *B. methanolicus* to create plasmid pTH1p12-*sfGFP* through assembly with PCR-amplified vector backbone
PROM31	TCGCTTATTTTCAAAAGATTATACTATCATA AAATTTCGGTTTTTTCAT	Forward primer for amplification of promoter (p15) derived from *B. methanolicus* to create plasmid pTH1p15-*sfGFP* through assembly with PCR-amplified vector backbone
PROM32	CTATTTATGTAATTGTTTACTTTTAAATTAT ATCACTACAAAAAGAGAGTACAA	Reverse primer for amplification of promoter (p15) derived from *B. methanolicus* to create plasmid pTH1p15-*sfGFP* through assembly with PCR-amplified vector backbone
PROM35	TCGCTTATTTTCAAAAGATTGGAAGTTTTTA TCTATCTACAACTTGAA	Forward primer for amplification of promoter (p17) derived from *B. methanolicus* to create plasmid pTH1p17-*sfGFP* through assembly with PCR-amplified vector backbone
PROM36	CTATTTATGTAATTGTTTACAATCATTATTG TATATAAATTAAAAGTATTTTTCTATTAC	Reverse primer for amplification of promoter (p17) derived from *B. methanolicus* to create plasmid pTH1p17-*sfGFP* through assembly with PCR-amplified vector backbone
PROM37	TCGCTTATTTTCAAAAGATTGTAAACCTTC AACTGAATAAGTTCGGTTATATCC	Forward primer for amplification of promoter (p18) derived from *B. methanolicus* to create plasmid pTH1p18-*sfGFP* through assembly with PCR-amplified vector backbone
PROM38	CTATTTATGTAATTGTTTACTGTATTATTTTA ACATATTGATGGGAAGCTGGCCTGA	Reverse primer for amplification of promoter (p18) derived from *B. methanolicus* to create plasmid pTH1p18-*sfGFP* through assembly with PCR-amplified vector backbone
PROM39	TCGCTTATTTTCAAAAGATTAAAGTTCTAG TTAATAAATGAGTTTTAAT	Forward primer for amplification of promoter (p19) derived from *B. methanolicus* to create plasmid pTH1p19-*sfGFP* through assembly with PCR-amplified vector backbone
PROM40	CTATTTATGTAATTGTTTACACTATAAAATA TGTTTCTAAAAAGAAATC	Reverse primer for amplification of promoter (p19) derived from *B. methanolicus* to create plasmid pTH1p19-*sfGFP* through assembly with PCR-amplified vector backbone
PROM41	TCGCTTATTTTCAAAAGATTAATAGATTAC GGGAACCGTCAT	Forward primer for amplification of promoter (p20) derived from *B. methanolicus* to create plasmid pTH1p20-*sfGFP* through assembly with PCR-amplified vector backbone
PROM42	CTATTTATGTAATTGTTTACCGTCCTAATAT TTCGACAAAAGGAG	Reverse primer for amplification of promoter (p20) derived from *B. methanolicus* to create plasmid pTH1p20-*sfGFP* through assembly with PCR-amplified vector backbone
LR11	GTAGTTGAATAATGATTCCTCATCAG	Forward primer for amplification of vector backbone pTHmplrBM-*sfGFP* for creation of constructs pTH1p01lrBM-*sfGFP*, pTH1p05lrBM-*sfGFP*, pTH1p12lrBM-*sfGFP* and pTH1p18lrBM-*sfGFP*. PROM02 used as reverse primer
PROM03	TCGCTTATTTTCAAAAGATTTATTTCTTTCC CTTTAAACTT	Forward primer for amplification of promoter p01 derived from *B. methanolicus* to create plasmid pTH1p01lrBM-*sfGFP* through assembly with PCR-amplified vector backbone. LR12 used as reverse primer

LR12	AGGAATCATTATTCAACTACACTGTTAATA TACTATCTATCA	Reverse primer for amplification of promoter p01 derived from *B. methanolicus* to create plasmid pTH1p01lrBM-*sfGFP* through assembly with PCR-amplified vector backbone. PROM03 used as forward primer
LR20	AGGAATCATTATTCAACTACAACTATTATT TTAGTTTTCAAAAAT	Reverse primer for amplification of promoter p01 derived from *B. methanolicus* to create plasmid pTH1p05lrBM-*sfGFP* through assembly with PCR-amplified vector backbone. PROM11 used as forward primer
LR14	AGGAATCATTATTCAACTACCTTTCAAATA TAGACCTTTTCAC	Reverse primer for amplification of promoter p01 derived from *B. methanolicus* to create plasmid pTH1p12lrBM-*sfGFP* through assembly with PCR-amplified vector backbone. PROM25 used as forward primer
LR15	AGGAATCATTATTCAACTACTGTATTATTTT AACATATTGATGGGAAGCTGGCCTGA	Reverse primer for amplification of promoter p01 derived from *B. methanolicus* to create plasmid pTH1p18lrBM-*sfGFP* through assembly with PCR-amplified vector backbone. PROM37 used as forward primer
PGF2	GTAAACAATTACATAAATAGGAGGTAGT	Forward primer for amplification of vector backbone pTHmp-*sfGFP* for creation of construct pTH1mpApr-*sfGFP*. PSGR used as reverse primer
APR01	GATTAGCATTTAAACTAGTTTTGGAAACGA ATCAATTAAATA	Forward primer for amplification of *B. subtilis*-derived *pbuE* riboswitch to create plasmid pTH1mpApr-*sfGFP* through assembly with PCR-amplified vector backbone.
APR02	CTATTTATGTAATTGTTTACTTTACTTAAAT GTTTTGATAAAT	Reverse primer for amplification of *B. subtilis*-derived *pbuE* riboswitch to create plasmid pTH1mpApr-*sfGFP* through assembly with PCR-amplified vector backbone.
APR03	ACTGTTAATATACTATCTATCA	Reverse primer for amplification of promoter p01 derived from *B. methanolicus* to create plasmid pTH1p01Apr-*sfGFP* through assembly with PCR-amplified *B. subtilis*-derived *pbuE* riboswitch and vector backbone. PROM03 used as forward primer.
APR04	ATAGATAGTATATTAACAGTGGAAACGAA TCAATTAAATA	Forward primer for amplification of *B. subtilis*-derived *pbuE* riboswitch to create plasmid pTH1p01Apr-*sfGFP* through assembly with PCR-amplified promoter p01 derived from *B. methanolicus* and vector backbone. APR02 used as reverse primer
APR05	AAATAGCTATTAAATTTTGTATAACCTC	Forward primer for site directed mutagenesis to introduce mutation in P1 region of *B. subtilis*-derived *pbuE* riboswitch. pTH1mpApr-*sfGFP* used as template.
APR06	GAGGTTATACAAAATTTAATAGCTATTT	Reverse primer for site directed mutagenesis to introduce mutation in P1 region of *B. subtilis*-derived *pbuE* riboswitch. pTH1mpApr-*sfGFP* used as template

Supplementary Table S2. Relative mean sfGFP fluorescence in pellets of *B. methanolicus*, *B. subtilis*, or *E. coli* recombinant strains carrying empty vector (pTH1mp), vector with *sfGFP* gene under the control of the P_{mdh} (pTH1mp-*sfGFP*) and vector with *sfGFP* gene under the control of the P_{mdh} and lysine riboswitch (pTH1mplrBM-*sfGFP*). OD_{600} normalised fluorescence from strains carrying the empty vector and vector pTH1mp-*sfGFP* are used as controls. OD_{600} normalised sfGFP fluorescence from cultures without or with 20 mM lysine added is shown (raw data for Figure 4).

**Strain**	**Plasmid**	**sfGFP fluorescence [FU/OD_{600}]**	
		**0 mM L-lysine**	**20 mM L-lysine**
Bacillus methanolicus	pTH1mp	509±40	459±36
	pTH1mp-*sfGFP*	25387±338	19109±541
	pTH1mplrBM-*sfGFP*	7157±342	1054±79
Bacillus subtilis	pTH1mp	87±11	78±11
	pTH1mp-*sfGFP*	51698±1169	65639±2988
	pTH1mplrBM-*sfGFP*	3606±45	1442±33
Escherichia coli	pTH1mp	250±19	248±25
	pTH1mp-*sfGFP*	2294092±44389	2375738±85985
	pTH1mplrBM-*sfGFP*	124424±3531	82622±1396

Supplementary Table S3. Effect of different amino acids on the activity of the lysine riboswitch. OD600 normalised sfGFP fluorescence from cultures without addition of amino acid or with 10 mM L-lysine, cadaverine, L-methionine, L-ornithine or L-proline is shown. The standard deviation of technical triplicates is shown.

	**MGA3(pTH1mp-*sfGFP*)**		**MGA3(pTH1mplrBM-*sfGFP*)**	
**Condition**	**sfGFP fluorescence [FU/OD_{600}]**	**Ratio to control condition**	**sfGFP fluorescence [FU/OD_{600}]**	**Ratio to control condition**
No supplementation (control)	32009±675	1.00	6492±258	1.00
10 mM L-lysine	27697±652	0.87	876±38	0.13
10 mM cadaverine	38275±879	1.20	6922±566	1.07
10 mM L-methionine	26893±559	0.84	4521±129	0.70
10 mM L-ornithine	31612±823	0.99	8321±173	1.28
10 mM L-proline	35608±486	1.11	7209±265	1.11

Supplementary Table S4. L-lysine final titers for MGA3 and M168-20 strains carrying different plasmids for controlled sfGFP production. The means of triplicates with standard deviations are shown.

**Strain**	**Plasmid**	**L-lysine titer [mg L^{-1}]**
Bacillus methanolicus MGA3	pTH1mp	0.2±0.0
	pTH1mp-*sfGFP*	0.3±0.0
	pTH1mplrBM-*sfGFP*	0.3±0.0
Bacillus methanolicus M160-20	pTH1mp	20.1±2.6
	pTH1mp-*sfGFP*	33.2±1.3
	pTH1mplrBM-*sfGFP*	37.2±3.0
